# On the *Campylobacter* Papers in this Issue

**DOI:** 10.14252/foodsafetyfscj.D-21-00014

**Published:** 2021-09-24

**Authors:** Shigeki Yamamoto

**Affiliations:** Food Safety Commission, Cabinet Office, Government of Japan, 5-2-20 Akasaka, Minato-ku, Tokyo 107-6122, Japan.

In the latest couple of years, people’s awareness regarding public health, microbial hazard readiness, and general hygiene has dramatically changed due to the advent of new corona virus infection. In Japan, the first case of new corona virus infection was officially confirmed on January 16, 2020 (IASR, Vol.42, No.2, 1-4, 2021), and the infection ever since has been defiantly persistent in this country despite the all-out efforts of prevention, including hand washing, disinfectant use, and vaccination. A total of four states of emergency have been declared between April 2020 and August 2021, in an attempt to change people’s behavioral habits of dining and gathering. In the meantime, looking at the numbers and proportion of food poisoning outbreaks during this period in contrast to the pre-COVID19 era, the numbers of food poisoning incidents and cases in year 2020 were 887 and 14,613, showing the down-ward trend compared to those of 1,061 and 13,081 observed in year 2019. Notably, the incident number decreased by 16% year on year in 2020. Although the number of patients increased, this may stem from larger-scale food poisoning incidents seen in the recent years. For example, the large-scale food poisonings due to *Escherichia coli* were reported from January 3 to July 12, 2021, resulting in 198 incidents affecting 2,286 patients according to the Ministry of Health, Labour and Welfare.

Although a clear connection between new corona virus pandemic and shift in frequency of food poisoning is yet to be studied, it is a plausible hypothesis that peoples’ behavioral change in hand washing, general awareness of sanitation and decreased opportunities of eating-out may directly or indirectly influenced the occurrence of food poisoning. 

*Campylobacter jejuni*/*coli* were the most common causes of food poisoning in Japan, while norovirus is the most common virus to cause food poisoning. In addition, Anisakis food poisoning is increasing in recent years. *Campylobacter *food poisoning accounts for 182 incidents (901 cases) in year 2020, also showing a down-ward trend from the previous year’s 286 incidents (1,973 cases). For year 2021, so far 33 incidents (192 cases) were reported till July. As *Campylobacter* food poisoning remains the most common among bacterial food poisonings in Japan, the recent drop in numbers of incidents/cases in *Campylobacter *food poisoning is an encouraging news in food safety field. Yet, its relations with cooking and eating practice modifications, including those brought by the pandemic, is worthy looking into in the future to further effectively manage and curve this food poisoning. Moving forward, the journal hopes the insights gained from the articles in this issue, among others, will help to shape food safety procedures in this trajectory.

